# Supplementary material for: Dominant effects of the immediate environment on the gut microbiome of mice used in biomedical research
Source: mSystems. 2025 Nov 12;10(12):e01112-25. doi: 10.1128/msystems.01112-25 (PMC12710307; doi:10.1128/msystems.01112-25)
Supplement: Supplemental figures — Figures S1 through S8. [file msystems.01112-25-s0001.pdf]

Supplementary Figures

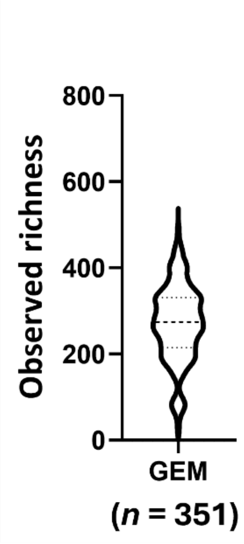

Figure S1. Violin plot showing distribution of observed richness in samples from GEM lines submitted to the MU MMRRC (*n* = 351 samples).

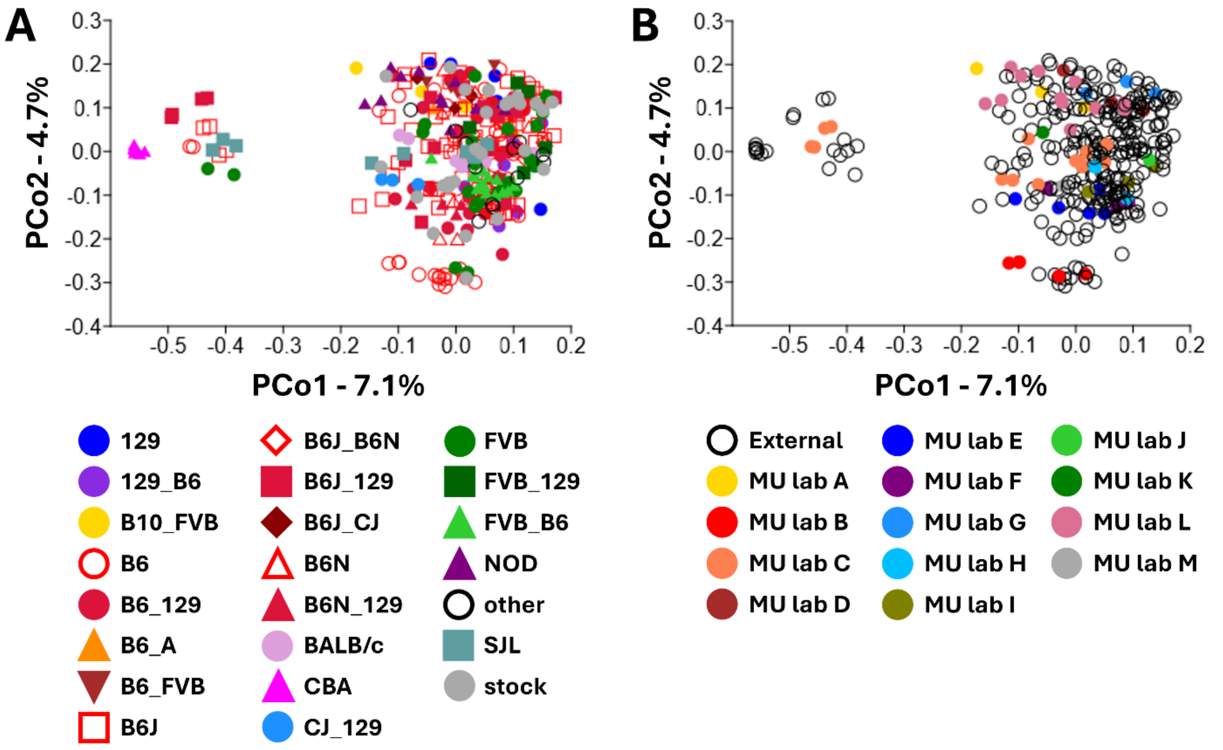

Figure S2. (**A**, **B**) Principal coordinate analysis (PCoA) plots showing unweighted beta-diversity among samples from GEM mice (*n* = 351) colored and/or shaped according to genetic background (**A**) or laboratory that generated and submitted the mice (**B**), legends below each plot. To reduce the number of groups in **A**, congenic strains and strains with mixed genetic background are combined using an underscore (e.g., B6.129 and B6;129 both counted as B6_129) and all 129 lines (e.g., 129S1, 129P2) are combined and counted as 129. To reduce the number of groups in **B**, GEMs submitted from each of 13 different labs (A through M) at the University of Missouri (MU) are colored by lab, and all GEMs submitted to the MU Mutant Mouse Resource and Research Center from external (non-MU) institutions are denoted by an open circle.

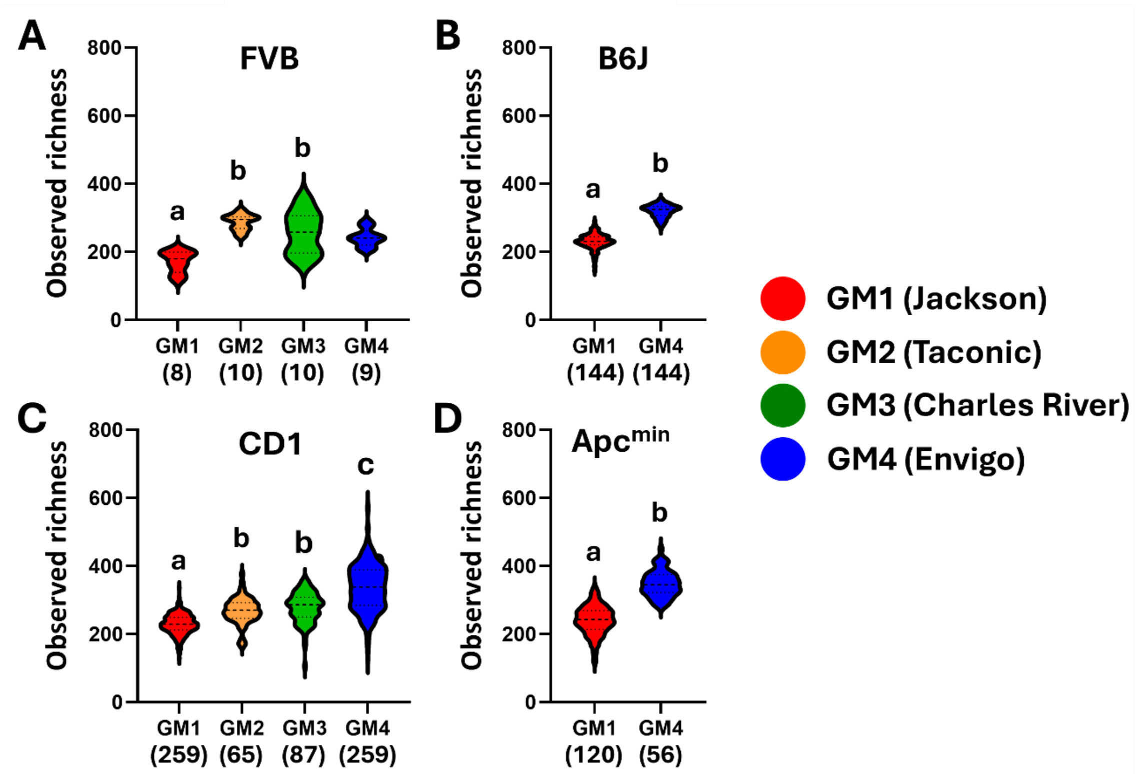

Figure S3. Violin plots showing distribution of observed richness in fecal samples from FVB (**A**), B6J (**B**), CD-1 (**C**), and Apc^{min} (**D**) mice (*n* below each group) colonized with different supplier-origin (SO) gut microbiomes (GM1 to GM4, origin of each in legend at right). Different letters indicate significant differences between SO GMs in one-way ANOVA, all $p < 0.05$.

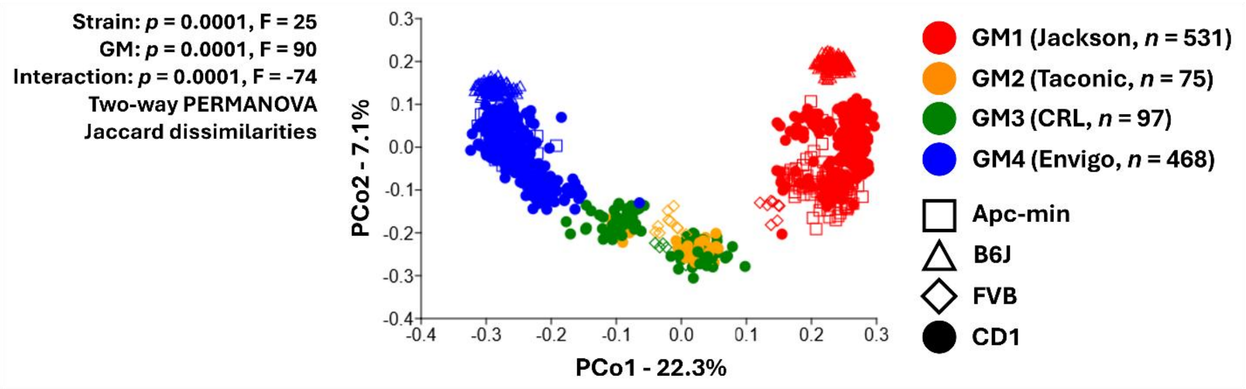

Figure S4. Principal coordinate analysis (PCoA) plots showing unweighted beta-diversity among samples from supplier-origin (SO) gut microbiome (GM)-colonized mice (*n* = 1,171, legend at right), as determined using amplicon sequence variant (ASV)-level features, colored according to SO GM and shaped according to genetic background. *p* and F values from two-way permutational multivariate analysis of variance (PERMANOVA) using Jaccard dissimilarities.

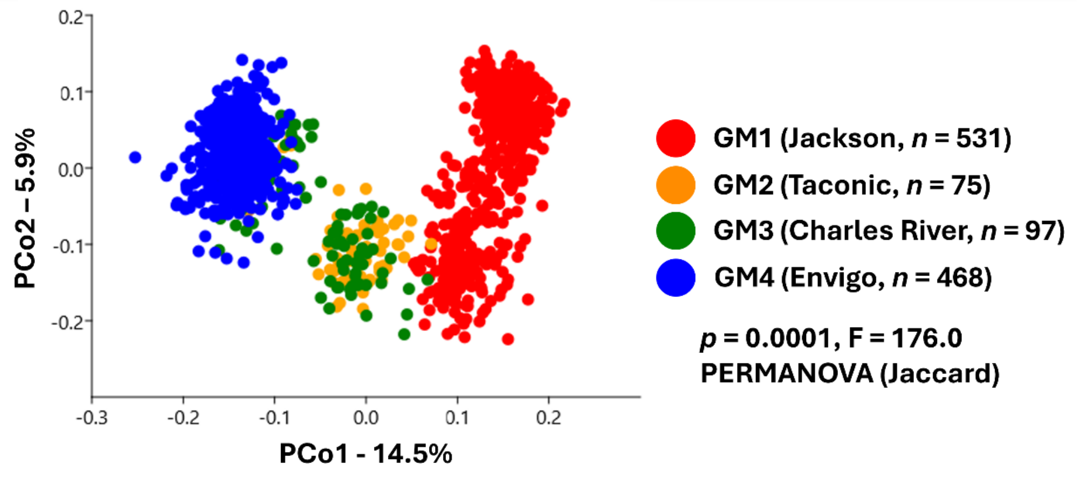

Figure S5. Principal coordinate analysis (PCoA) plots showing unweighted beta-diversity among samples from supplier-origin (SO) gut microbiome (GM)-colonized mice (*n* = 1,171, legend at right), as determined using features collapse to the level of genus. *p* and F values from one-way permutational multivariate analysis of variance (PERMANOVA) using Jaccard dissimilarities.

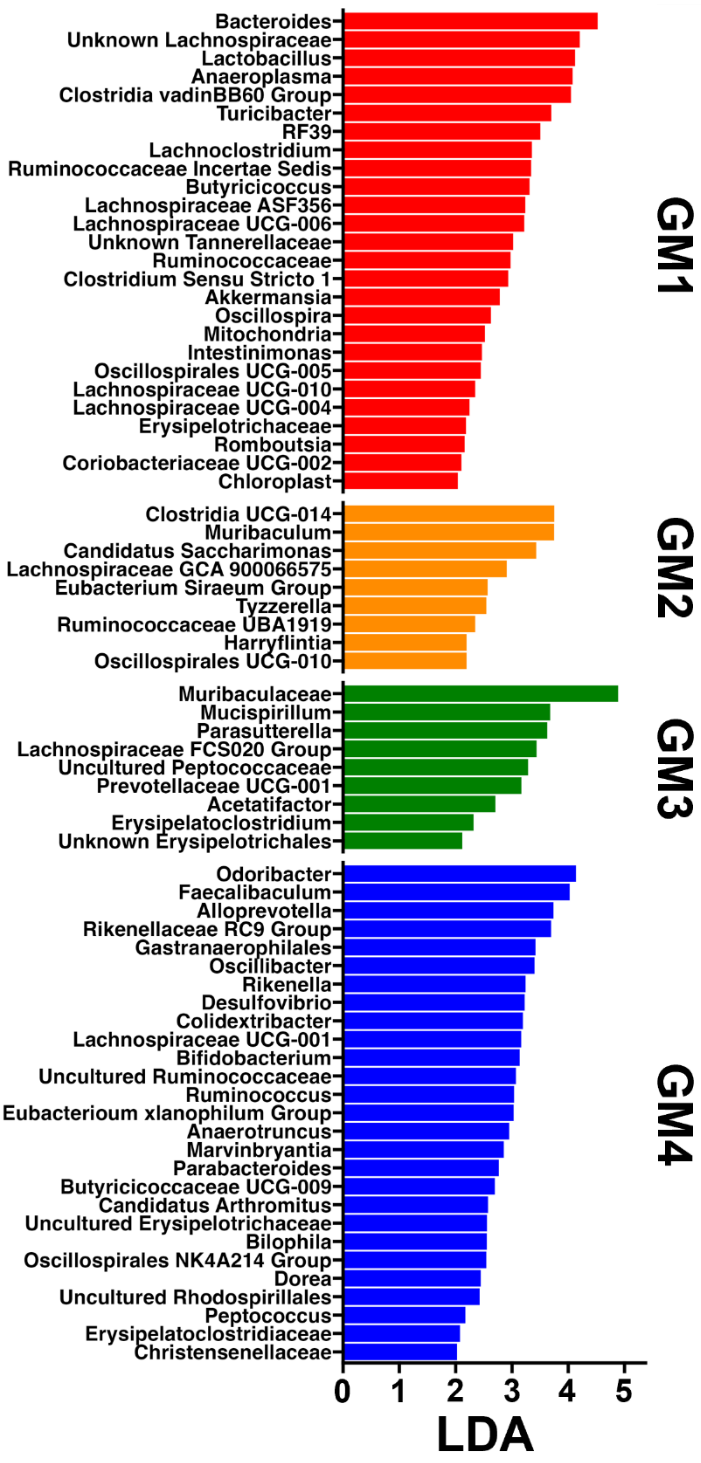

Figure S6. Bar chart showing results of linear discriminant analysis (LDA) effect size (LEfSe) analysis, indicating genera associated with each of the four supplier-origin gut microbiomes.

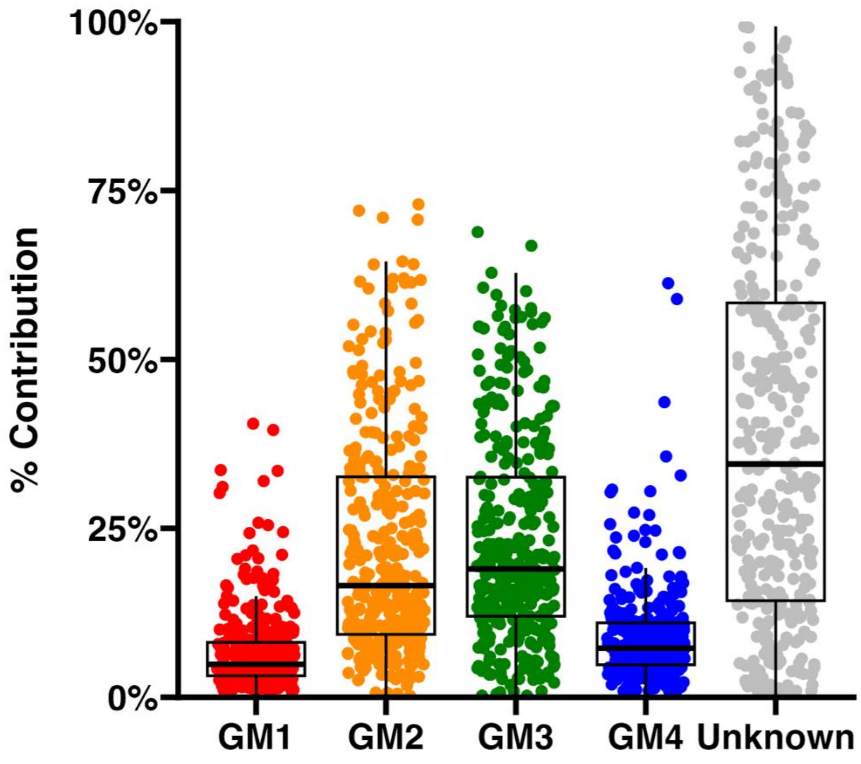

Figure S7. Box plots showing percent contribution to GEM samples (n = 351) from each SO GM and from unknown sources.

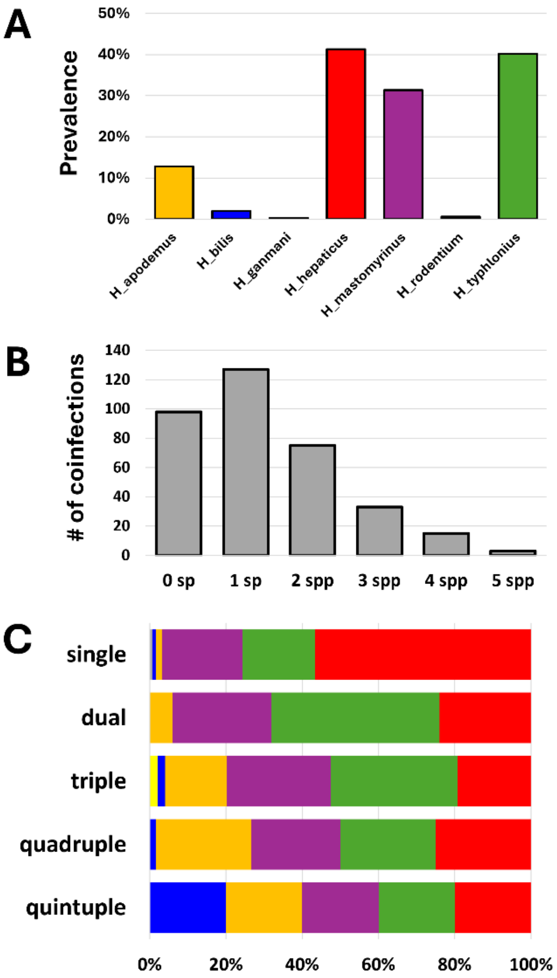

Figure S8. (**A**) Bar chart showing the prevalence of each *Helicobacter* sp. among GEMs (*n* = 351), based on 16S rRNA metabarcoding data. (**B**) Bar chart showing the prevalence among GEM samples (*n* = 351) of 0, 1, or more *Helicobacter* spp. detected in the same sample and (**C**) pie charts showing the representation of each Helicobacter sp. among single and multi-species colonization.
